# The association between health professionals’ international experience and the academic output of their students in Harbin, China

**DOI:** 10.1186/s12909-019-1853-y

**Published:** 2019-11-20

**Authors:** Tingjiao Liu, Liming Zhang, Tingting Zhao, Nan Chen

**Affiliations:** Department of Neurology, The First Affiliated Hospital of Harbin Medical University, Harbin, China

**Keywords:** International experience, Research capacity, Chinese medical student, Advisor

## Abstract

**Background:**

It is common practice for health professionals in China to have international experience. However, the association between such experience and these professionals’ students’ scientific research ability has not previously been evaluated. Our study aimed to quantify this association among the students of health professionals in China.

**Methods:**

We constructed a self-administered questionnaire and distributed it to all students at Harbin Medical University and its affiliated hospitals, including 257 students (Group A) of health professionals who had studied overseas (“returning” professionals) and 257 age-, enrollment year-, and specialty-matched students (Group B) of health professionals who had not studied overseas (“resident” professionals). SPSS software was used for the data entry and analysis.

**Results:**

The total impact factor (IF) for articles published during their PhD study was 1031.68 in Group A and 727.65 in Group B (*P* = 0.001), and the number of articles was 297 in Group A and 228 in Group B (*P* = 0.040). The total IF for articles published by the 151 clinical medicine students of returning professionals during their PhD study was positively correlated with their advisor’s total IF for articles published while abroad (*P* = 0.019).

**Conclusions:**

This study indicates that medical students may benefit from their advisors’ international experience. Medical education administrators and the government could encourage clinical professionals to study overseas and to prolong the duration of their study abroad. Medical students should consider potential advisors’ overseas experience when choosing a mentor.

## Background

Over the past decade, there has been an increasing emphasis among university educators on globalization and internationalization, and global health programs and international experience have become key areas of focus for university professionals. According to China’s Ministry of Education, in 2017, the number of Chinese students studying abroad exceeded 600,000 for the first time, reaching 608,400, which was an increase of 11.74% compared with 2016 [1]. Researchers may benefit from experiencing the cultural differences inherent in exchange programs, and they may bring an awareness of these differences into their curricula, thereby broadening international training opportunities through work, education, and research activities [2–7]. After returning home, many health professionals work at medical universities or affiliated hospitals. As an implicit responsibility of these roles, health professionals must mentor medical students through engagement in clinical work and scientific research. The research ability of medical students during their postgraduate studies depends on many aspects, including the background and extent of their advisors’ education and scientific research ability. Our previous research [8] has shown that overseas study experience improved health professionals’ scientific research ability, but no previous research has examined the impact of overseas experience on those professionals’ students’ scientific research ability. Is there a gap in the research ability of students of health professionals who studied or trained overseas (“returning” professionals) and that of students of health professionals who did not train overseas (“resident” professionals)? Do returning professionals, as postgraduate advisors, have a greater positive impact on their students’ scientific research ability, compared with resident professionals? Does overseas experience play a positive role in the development of the research ability of medical students in China? In this study, we set out to answer these questions and to encourage the construction of a health professionals’ overseas-experience database to help postgraduate students choose highly effective advisors. We also hope that our research results will be helpful in forming better training programs for medical students and in improving medical education policy.

## Methods

Harbin Medical University (HMU) is the only Western medical institution in Harbin, in China’s Heilongjiang Province. It is a government institution that offers a five-year medical bachelor’s degree course, a three-year master’s degree course, and a three-year PhD degree course. HMU has recently begun to restructure and reform its medical education program to offer comprehensive solutions to national medical education problems.

This study was conducted from September 2016 to April 2018. During this period, only 1–2% of master’s students (*n* = 1561) published a Science Citation Index (SCI) article prior to graduation. In contrast, around 70% of PhD students (*n* = 1083) published SCI articles before graduating. Thus, we included only PhD students in our analyses. PhD students in China are differentiated by their year of enrollment. PhD students in the class of 2015 graduated in June 2018 and their articles may not have been published by the end of the research period, so we selected PhD students enrollment in 2012–2014 for our study. Health professionals with a minimum of 6 months study-abroad experience were categorized as “returning,” because we designated 6 months as the minimum experience required to be an independent researcher. Therefore, health professionals with less than 6 months study-abroad experience were excluded from this study. In the study, both “returning” and “resident” professionals had PhD degrees. These professionals were 56 scientific researchers from HMU and 76 clinical doctors from HMU’s affiliated hospitals.

We analyzed 257 students of returning health professionals (Group A)-106 from HMU (Group A_1_), who were trained to be scientific researchers, and 151 from HMU’s affiliated hospitals (Group A_2_), who were trained to be clinical doctors. To collect information effectively, we constructed the questionnaire presented in Additional file 1 Table S1, which was distributed to each of the 257 students. To ensure confidentiality and a high response rate, three investigators meet the students face to face and distributed the questionnaires to them. The students were required to complete the questionnaires while the investigator was present, and the investigators were not allowed to disclose any information or data collected.

The SCI is internationally recognized as an authoritative scientific literature search tool. We used the SCI impact factor (IF) as an indicator of scientific and research capability. Because authorship contribution is determined differently across institutions and countries and there is no international system to weigh these differences against, we ranked the authors to reflect contribution differences according to HMU’s 2013 official promotion system. This system uses the following formulae: first author or corresponding author = IF * 100%; second author = IF * 50%; and third or later author = IF * 25% [9].

We compared the publishing histories of the students of returning professionals with those of the students of resident professionals to estimate the impact of advisors’ overseas experience on their students’ research capacity. Other relevant advisor information was included, such as total IF, number of articles published while abroad, duration of overseas study, and age at travel abroad. We tried to find out the correlations between these overt factors and students’ scientific research ability and identify which factor should be considered most for students when choosing advisors.

The following data were collected from 257 students of returning professionals: (1) student’s and advisor’s names; (2) student’s and advisor’s ages; (3) student’s and advisor’s sexes; (4) student’s enrollment year (Grade); (5) student’s school and department; (6) student’s total IF for articles published during their PhD study; (7) student’s number of articles published during their PhD study; (8) advisor’s duration of study or training abroad (months); (9) advisor’s age when they went abroad; (10) advisor’s total IF for articles published while abroad; and (11) advisor’s number of articles published while abroad. We used SPSS, Version 19 (IBM Corp., Armonk, NY) to estimate multiple linear regression models to identify the factors associated with student’s IF for articles and with the number of articles students published during their PhD study. To explore the relationship between health professionals’ international experience and the academic output of their students, we selected 257 age-, enrollment year-, and specialty-matched students of resident professionals (Group B)—106 scientific research students from HMU (Group B_1_) and 151 clinical medicine students from affiliated hospitals (Group B_2_). We compared the total IF and the number of articles published between the returning and resident groups. We selected the control group (Group B) based on exact matches for age, year, and specialty with Group A participants. And there were 78 students of Group A who did not have a match. In total, there were 694 students enrolled in 2012–2014, 335 of whom were students of returning professionals. To avoid arbitrarily selecting matching controls, when there was more than one match, we randomly selected one control, without considering student’s research backgrounds and advisor’s name. We replaced study cases for whom we could not find an exact match with another student who had at least one match. We distributed the same questionnaires to the 257 control students of resident professionals, and questions regarding the advisor’s information of studying abroad were not filled.

## Results

In this study, the questionnaire response rate is 100%. Of the 257 students of returning professionals (Table 1, Group A), there were 82 students in the 2012 graduating class, 87 in the 2013 class, and 88 in the 2014 class. Students’ ages ranged from 26 to 34 years (mean = 31.29 years). In Group A, there were 106 scientific research students from HMU (Group A_1_: 37 students in the 2012 class, 32 students in the 2013 class, and 37 students in the 2014 class), with an age range of 26–34 years (mean = 31.32 years), and 151 clinical medicine students from affiliated hospitals (Group A_2_: 45 students in the 2012 class, 55 students in the 2013 class, and 51 students in the 2014 class), with an age range of 26–34 years (mean, 31.26 years). Similarly, there were 257 age-, enrollment year-, and specialty-matched students of resident professionals with the same grade and age proportion as those in Group A (Table 2, Group B), consisting of 106 scientific research students (Group B_1_: 37 students in the 2012 class, 32 students in the 2013 class, and 37 students in the 2014 class), with an age range of 26–34 years (mean = 31.32 years), and 151 clinical medicine students (Group B_2_: 45 students in the 2012 class, 55 students in the 2013 class, and 51 students in the 2014 class), with an age range of 26–34 years (mean = 31.26 years).

**Table 1:** Data of 257 students of returning professionals (Group A), including 106 scientific research students (Group A_1_) and 151 clinical medicine students (Group A_2_)

	Maximum	Minimum	Mean
Total IF of papers published during PhD	Group A 58.23	Group A 0	Group A 4.01
	Group A_1_ 58.23	Group A_1_ 0	Group A_1_ 4.17
	Group A_2_ 26.82	Group A_2_ 0	Group A_2_ 3.91
Number of papers published during PhD	Group A 10	Group A 0	Group A 1.16
	Group A_1_ 10	Group A_1_ 0	Group A_1_ 1.15
	Group A_2_ 4	Group A_2_ 0	Group A_2_ 1.16
Tutor’s duration of studying abroad (months)	Group A 192	Group A 6	Group A 38.54
	Group A_1_ 192	Group A_1_ 6	Group A_1_ 42.16
	Group A_2_ 120	Group A_2_ 12	Group A_2_ 36
Tutor’s age of going abroad	Group A 46	Group A 30	Group A 36.39
	Group A_1_ 46	Group A_1_ 32	Group A_1_ 36.51
	Group A_2_ 45	Group A_2_ 30	Group A_2_ 36.30
Tutor’s total IF of papers published while abroad	Group A 94.69	Group A 0	Group A 7.59
	Group A_1_ 94.69	Group A_1_ 0	Group A_1_ 11.35
	Group A_2_ 60.22	Group A_2_ 0	Group A_2_ 4.94
Tutor’s number of papers published while abroad	Group A 30	Group A 0	Group A 2.58
	Group A_1_ 30	Group A_1_ 0	Group A_1_ 4.17
	Group A_2_ 13	Group A_2_ 0	Group A_2_ 1.46

**Table 2:** Data of 257 students of resident professionals (Group B), including 106 scientific research students (Group B_1_) and 151 clinical medicine students (Group B_2_)

	Maximum	Minimum	Mean
Total IF of papers published during PhD	Group B 21.66	Group B 0	Group B 2.83
	Group B_1_ 21.66	Group B_1_ 0	Group B_1_ 2.71
	Group B_2_ 12.13	Group B_2_ 0	Group B_2_ 2.92
Number of papers published during PhD	Group B 5	Group B 0	Group B 0.89
	Group B_1_ 5	Group B_1_ 0	Group B_1_ 0.76
	Group B_2_ 4	Group B_2_ 0	Group B_2_ 0.97

**Fig. 1:**
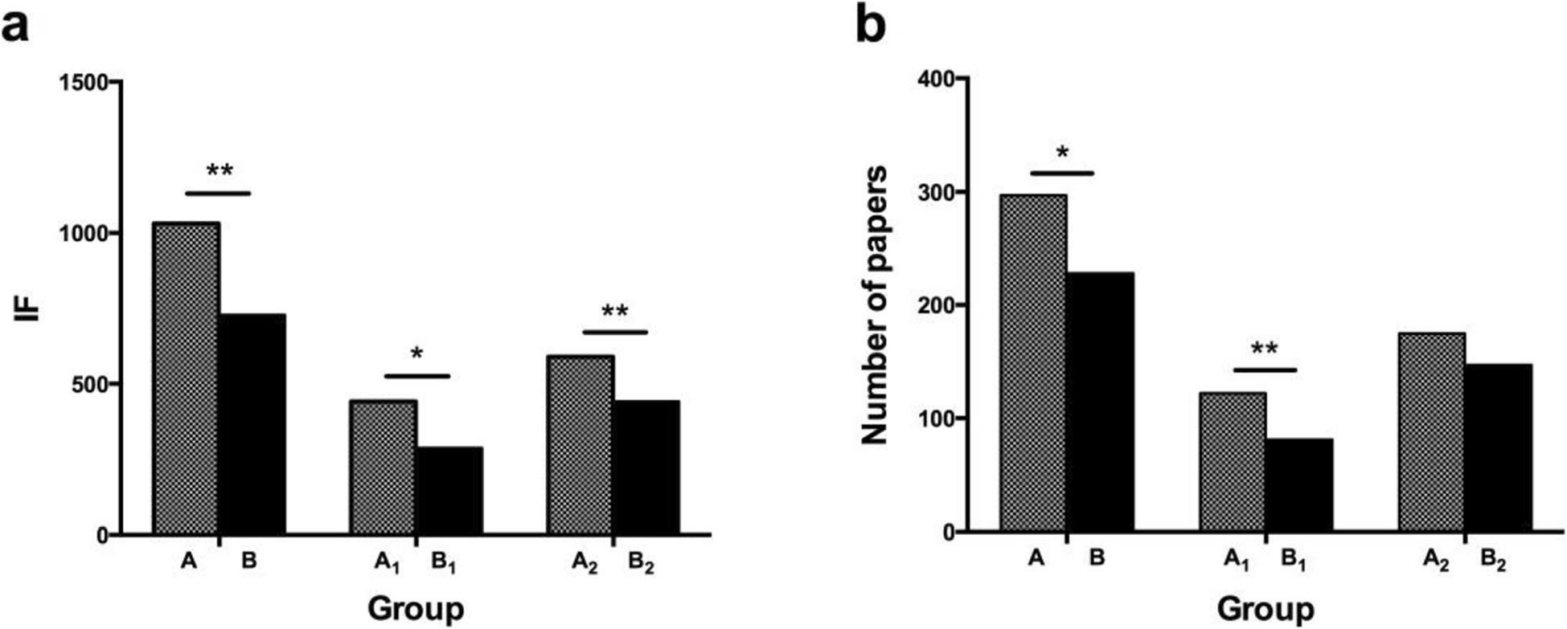
Total IF and number of papers of returning professionals’ students and resident professionals’ students during their PhD. **a** Total IF of returning professionals’ students and resident professionals’ students. **b** Number of papers of returning professionals’ students and resident professionals’ students. Group A/A_1_/A_2_: returning professionals’ students; Group B/B_1_/B_2_: resident professionals’ students. For 3 paired samples (Group A and Group B, Group A_1_ and Group B_1_ and Group A_2_ and Group B_2_) statistical differences were found for total IF of papers (**P < 0.01, *P < 0.05) using a t -test. But for number of papers statistical differences (**P < 0.01, *P < 0.05) were only found for 2 paired samples (Group A and Group B and Group A_1_ and Group B_1_) using a t -test

The IF for articles published by individual students of returning professionals during their PhD study (Table 1, Group A) ranged from 0.00 to 58.23 (mean = 4.01), and the number of articles ranged from 0 to 10 (mean = 1.16). The total IF for articles published during PhD study in Group A was 1031.68, and the total number of articles was 297. The total IF for articles published during PhD study was 441.61 in Group A_1_ and 590.07 in Group A_2_. The number of articles was 122 for Group A_1_ and 175 for Group A_2_. In contrast, the individual IF for resident professionals (Table 2, Group B) ranged from 0.00 to 21.66 (mean = 2.83), and the number of articles ranged from 0 to 5 (mean = 0.89). The total IF for articles published in Group B was 727.65, and the total number of articles was 228. A wide gap was observed between Group A and Group B for both total IF and number of articles. The total IF for articles published was 286.79 in Group B_1_ and 440.86 in Group B_2_. The number of articles was 81 in Group B_1_ and 147 in Group B_2_. We compared the mean scores using a *t*-test for three paired samples (Group A and Group B, Group A_1_ and Group B_1_, and Group A_2_ and Group B_2_; Fig. 1). We found statistically significant differences between Group A and Group B for total IF (*P* = 0.001) and total number of articles (*P* = 0.040). There were significant differences in the mean scores for total IF (*P* = 0.040) and the total number of articles (*P* < 0.001) between Group A_1_ and Group B_1_. Group A_2_ and Group B_2_ had significantly different mean scores for total IF (*P* = 0.009), but the difference between these groups for the total number of articles (*P* = 0.061) was not statistically significant.

Given the results of our multiple linear regression analysis of all 257 students of returning professionals (Table 1), we concluded that the IF earned during a student’s PhD study was not related to their advisor’s study-abroad duration (*P* = 0.719), their advisor’s age when they studied abroad (*P* = 0.088), or their advisor’s number of articles published while abroad (*P* = 0.843). However, the total IF for articles published by the 151 clinical medicine students of returning professionals during their PhD study was positively correlated with their advisor’s total IF for articles published while abroad (*P* = 0.019). A strong linear association was observed between the total IF for articles published by the clinical medicine PhD students of returning professionals and the total IF for their advisor’s articles published while abroad. However, for the full sample, the number of articles published by returning professionals’ students during their PhD study was not associated with their advisor’s study-abroad duration (*P* = 0.883), their advisor’s age when they studied abroad (*P* = 0.310), their advisor’s total IF for articles published while abroad (*P* = 0.408), or their advisor’s number of articles published while abroad (*P* = 0.880).

## Discussion

Engagement in international training experiences provides significant benefits to health professionals, including an appreciation of cultural diversity, the capacity to adapt to societal change, knowledge of alternative approaches to health and disease, and an understanding of public health and its implications for underserved populations. It is often assumed that a health professional’s study-abroad experience has a positive influence on the scientific research capacity of their students; however, such an influence had not previously been evaluated in China. Our study revealed that, for both scientific research students and clinical medicine students, there is a wide gap between the research abilities of students of returning professionals and students of resident professionals. These data strongly suggest that not only scientific research students but also clinical medicine students can benefit from their advisors’ international experience, presumably because their research capacity is indirectly elevated by their advisors’ broadened horizons. These findings suggest that advisors with overseas experience will cultivate more capable medical students.

How can China promote this positive influence? There is no precedent for establishing legislation or other official measures that incentivize medical trainees and professionals to gain research experience overseas [10]. It is important for clinical faculty, administrators, and government officials engaged in medical education to integrate resources into training programs that support advisors’ overseas training for the purpose of improving medical students’ research capacity. We found a significant relationship between the total IF for articles published by the clinical medicine PhD students of returning professionals and the total IF for advisor’s articles published while abroad. Furthermore, our previous work has shown that this relationship was tied to the advisor’s duration of study abroad but not to their age when they studied abroad [8]. In contrast, the total IF for articles published by the 106 scientific research students during their PhD study was not associated with their advisor’s total IF for articles published while abroad (*P* > 0.05).

On the basis of our findings, we conclude that medical education administrators and the government could encourage clinical professionals to study overseas and to prolong the duration of their study abroad to a minimum of 24 months to improve the IF of their work while overseas. For example, the China Scholarship Council, which currently requires traveling scholars to cover their own living and tuition expenses for prolonged overseas stays, could consider offering additional support for up to 2 years to the most outstanding scholars.

Another conclusion we drew from our findings is that medical students should consider their potential advisors’ overseas experience when choosing a mentor. Advisors with overseas experience tend to cultivate medical students with higher research ability.

Health professionals cannot produce articles worthy of a high IF without state-of-the-art laboratory equipment and facilities. Health professionals studying at overseas institutions have access to fully equipped and up-to-date laboratories that allow them to engage with advanced technology and move their research ideas forward. After spending time overseas, these professionals import these experiences and skills upon returning to China, and they incorporate them into their research and teaching. In contrast, the innovative thinking and research capacity of resident professionals is hampered by the limited infrastructure in China, possibly leading to resident professionals not cultivating an equivalent research capacity in their students, compared with returning professionals.

During our research with returning professionals and their students, many reported finding more innovative conditions and attitudes abroad, compared with Chinese institutions. This seemed to be the main factor that motivated them to study abroad. This difference is not only because of poor research conditions in China, but also because of insufficient investment in medical research. We argue that officials at higher levels of the political hierarchy should take bold steps to improve the national research capability and to cultivate more innovation among medical experts.

Besides, in the near future, we are going to do further research on how international experiences of advisors in English language countries versus non-English language countries, and low-resource environments versus high-resource environments, would impact the academic output of their students in order to find out the optimization training model for health professionals in China.

### Study limitations

Our work is an initial effort to better understand the impact of health professionals’ study-abroad experience on their PhD students’ research productivity, using medical institutions in Harbin as a case study. We were only able to recruit a small sample from a single medical school and its four affiliated hospitals; therefore, we cannot generalize our findings to the entire medical education situation in China. Additionally, because authors make non-uniform decisions about assigning authorship for research contributions and there is no international standard for ranking authors in a universal manner, we ranked authors using HMU’s promotion system (2013); however, this system cannot be applied to SCI articles worldwide. Furthermore, professionals who undertook 6 months or more of international training are likely to have high-level administrative titles, so more candidates apply for PhD positions with these professionals. Only one or two students can obtain PhD positions with each professional per year, so the students of these professionals are likely to be particularly competitive among their peers. Consequently, these students may have higher capability and motivation at baseline, when they enter the PhD program, so comparisons of students of returning and resident professionals may be biased unless the students’ capability to conduct research and write articles at baseline can be quantified and matched. Furthermore, professionals who are selected for overseas training may be more accomplished because of prior experience or articles, which may explain their selection for this type of training. This selection may therefore be a “marker” for their capabilities, which may have remained the same if they had not gone overseas, meaning that they would still have had a positive benefit on their students without overseas training. In addition, the data used in this study were collected using self-administered questionnaires, which may have omissions or inaccuracies, compared with independent observation of publications in the literature.

## Conclusions

This study suggests that the research ability of medical students in China is higher among those whose advisors studied abroad because the total IF for articles published by the clinical medicine PhD students of returning professionals was associated with their advisors’ total IF for articles published while abroad. Furthermore, returning clinical professionals can improve their total IF while overseas by prolonging their study-abroad duration, which will, in turn, benefit their future students. We suggest that medical students consider potential advisors’ overseas backgrounds when choosing a mentor. Finally, we identified a strong need to upgrade research facilities and to increase investment in medical research in China.

## Supplementary information

**Additional file 1: Table S1.** Questionnaire which was administered to 257 students of ‘returning’ professionals.

## Data Availability

The datasets used and analyzed for the current study are available from the corresponding author on reasonable request.

## Abbreviations

HMU: Harbin Medical University
IF: Impact factor
SCI: Science Citation Index
