# Supplementary material for: The association between health professionals’ international experience and the academic output of their students in Harbin, China
Source: BMC Med Educ. 2019 Nov 20;19:428. doi: 10.1186/s12909-019-1853-y (PMC6868785; doi:10.1186/s12909-019-1853-y)
Supplement: Supplementary file 1 — Additional file 1: Table S1. Questionnaire which was administered to 257 students of ‘returning’ professionals. [file 12909_2019_1853_MOESM1_ESM.docx]

**Table S1**

The questionnaire for students of Chinese health professionals in Harbin who have studied or trained abroad

Name: Age: Sex: Enrollment year (Grade):

School and Department:

Name of your tutor: Sex: Age:

For you:

1. How many scientific papers did you publish during your PhD?
2. What were the IFs of these papers?
3. Which ranked author were you?

For your tutor:

1. Studied or trained abroad from (year/month) to (year/month) in (country) University.

2. How many scientific papers did your tutor publish while abroad?

3. What were the IFs of these papers?

4. Which ranked author were your tutor?
